# Phosphoprotein Contributes to the Thermostability of Newcastle Disease Virus

**DOI:** 10.1155/2018/8917476

**Published:** 2018-11-04

**Authors:** Yang Zhao, Huairan Liu, Feng Cong, Wei Wu, Ran Zhao, Xiangang Kong

**Affiliations:** ^1^College of Veterinary Medicine, Northeast Agricultural University, No. 600 Changjiang Street, Xiangfang District, Harbin 150030, Heilongjiang Province, China; ^2^Division of Avian Infectious Diseases, National Key Laboratory of Veterinary Biotechnology, Harbin Veterinary Research Institute, The Chinese Academy of Agricultural Sciences, No. 678 Haping Road, Xiangfang District, Harbin 150069, China

## Abstract

Newcastle disease (ND), caused by Newcastle disease virus (NDV), is highly contagious and represents a major threat to the poultry industry. The thermostable vaccines are not insensitive to heat and ease of storage and transportation, but the mechanism of NDV thermostability remains unknown. The phosphoprotein (P), fusion protein (F), hemagglutinin-neuraminidase protein (HN), and large polymerase protein (L) are associated with NDV virulence. The association between F, HN, or L and viral thermostability has been, respectively, studied in different reports. However, the effects of P on NDV thermostability have not been demonstrated. Here, we utilized an existing reverse genetics system in our laboratory, to generate chimeric viruses by exchanging the P protein between the thermostable NDV4-C strain and the thermolabile LaSota strain. Chimeric viruses were found to possess similar growth properties, passage stability, and virulence, as compared to those of these parental strains. Interestingly, the thermostability of the chimera with P derived from the thermolabile LaSota strain was reduced compared to that of the parental virus, and P of the thermostable NDV4-C strain enhanced chimeric virus thermostability. Our data demonstrate that P is an important factor for the thermostability of NDV and provides information regarding the molecular mechanism of NDV thermostability; moreover, these results suggest a theoretical basis for using the NDV4-C strain as a thermostable vaccine.

## 1. Introduction

Newcastle disease (ND) is a highly contagious respiratory, enteric, or neurological disease of chickens caused by Newcastle disease virus (NDV), which infects more than 240 species of birds [1]. ND is a major threat to the poultry industry worldwide, causing significant economic losses, despite forced vaccination for ND in many countries [2, 3]. Most live conventional NDV vaccines need to be kept at low temperatures for preservation with cold chain transportation, which represents up to 80% of the entire cost of vaccination programs [4]. Meanwhile, the cold chain is affected by equipment and human factors, which means that the quality of vaccines cannot be guaranteed during their storage and transport [5–7]. Moreover, the processes of low temperature storage and cold chain transportation have always failed to achieve the desired protection of the vaccine [8, 9]. Hence, a thermostable NDV vaccine is required to overcome the downfalls of low temperature storage and cold chain transportation.

Many NDV vaccine strains are thermolabile, such as LaSota; however, few are thermostable, including the V4 strain. The V4 strain was isolated from the stomach of an 8-week-old chicken in Australia in 1966 and is avirulent and heat-resistant [10]. In our laboratory, we successively propagated this strain in embryonated specific-pathogen-free (SPF) chicken embryos for more than 20 generations to obtain the NDV4-C strain, which has retained the avirulent and thermostable characteristics of the parental V4 strain. Moreover, experiments using chickens demonstrated that NDV4-C immunogenicity is superior to that of the V4 strain [11, 12]. Therefore, NDV4-C is a good candidate for the development of a heat-resistant NDV vaccine.

NDV has a nonsegmented, single-stranded, negative-sense RNA genome consisting of six genes that encode at least seven proteins including the nucleocapsid protein (NP), phosphoprotein (P), matrix protein (M), fusion protein (F), hemagglutinin-neuraminidase protein (HN), and large polymerase protein (L). Existing reports have confirmed that P, F, HN, and L are associated with NDV virulence [13–16]. Among these, F, HN, and L have been studied regarding their relationship with virus thermostability. HN, rather than F and L, is a critical determinant of NDV thermostability [17]. However, the association between P and thermostability has not been studied and thus remains unclear.

Some characteristics and functions of P have been suggested by many reports. During P gene transcription, two additional nonstructural proteins, V and W, are produced via mRNA editing [18]. P has multiple roles and is vital for viral RNA synthesis [19, 20]. This protein forms complexes with NP and L, which confers tight encapsidation to NDV genomic RNA [21]. P interacts with NP to ensure that the latter exists in a soluble state and results in the production of RNA specific to the virus. Meanwhile, P binds to L, enhancing the interaction between L and NDV RNA templates [22].

In this study to investigate the effect of P on the thermostability of NDV, we used existing reverse genetic technology and the NDV4-C thermostable strain from our laboratory [11, 12]. We exchanged P proteins between the thermostable NDV4-C strain and the thermolabile LaSota strain; using these chimeras, we compared replication properties, passage stability, virulence, and thermostability with those of parental strains. Our data showed that P did not affect growth, stability, or virulence of NDV strains but played a key role in thermostability.

## 2. Material and Methods

### 2.1. Viruses and Cells

NDV4-C and LaSota strains were preserved in our laboratory. Viruses were propagated in 10-day-old chick embryo allantoic cavity and stored at −70°C. BHK-21 cells and BSR-T7/5 cells stably expressing T7 polymerase were all maintained in our laboratory and were grown in Dulbecco's modified Eagle's medium (DMEM, Thermo Scientific, Rockford, IL) with 10% fetal bovine serum (Gibco, Life Technologies, Australia) at 37°C and 5% CO_2_.

### 2.2. Construction of Full-Length Chimeric NDV4-C and LaSota Antigenomic cDNAs

**Figure 1:**
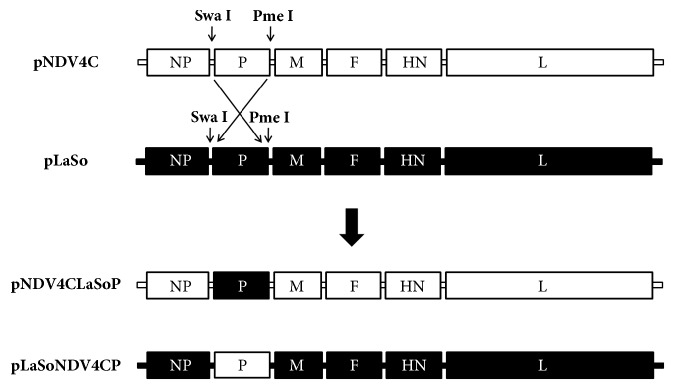
**Schematic representation of strategies to exchange the P gene between the pNDV4C and pLaSo vectors. **White and dark bars represent genes of the NDV4-C and LaSota strain, respectively. Unique restriction sites SwaI and PmeI were created on both pNDV4C and pLaSo cDNAs to exchange the P gene.

The construction of full-length antigenomic cDNAs of NDV4-C (pNDV4C) has been described previously [12]. A plasmid encoding the LaSota full-length infectious clone, pLaSo, and the helper plasmids pCI-NP, pCI-P, and pCI-L were generously donated by the Central Veterinary Institute of Wageningen UR, Netherlands. To exchange P genes between pNDV4C and pLaSo, we used unique restriction sites including SwaI in the 3′ untranslated region (UTR) of the NP gene and a PmeI in the 3′UTR of the P gene in the full-length cDNAs of both pNDV4C and pLaSo, according to a previous report [14] (Figure 1). The P gene open frame (ORF) was exchanged using the SwaI and PmeI sites. pNDV4C harboring the P gene of the LaSota strain (instead of its own P gene) was designated pNDV4CLaSoP, whereas pLaSo with the P gene of NDV4-C (instead of its own P gene) was designated pLaSoNDV4CP (Figure 1). All exchanged regions in full-length cDNA were sequenced to confirm that the desired gene replacement was achieved.

### 2.3. Rescue of Virus from cDNA

This procedure was performed to recover parental and chimeric NDVs according to previous reports [12]. BSR-T7/5 cells were grown to approximately 85% confluency in monolayers in six-well plates and gently washed three times with phosphate-buffered saline prior to transfection. A total of 10 *μ*g of the full-length plasmid, pCI-NP-K, pCI-P-K, and pCI-L-K were cotransfected into BSR-T7/5 cells using Lipofectamine™ 2000 (Invitrogen Carlsbad, CA, USA) at a ratio of 4:2:2:1. Then, 1 *μ*g/ml TPCK (L-1-tosylamide-2-phenylethyl chloromethyl ketone)-trypsin (Sigma) was added to the medium. After 3-4 days, the culture supernatants and cells were harvested and used to inoculate 9-day-old SPF chicken embryos until NDV-specific HA could be detected in the allantoic fluid.

### 2.4. Growth Characteristics and Passage Stability of Chimeric Viruses in SPF Chicken Embryos

To study the growth characteristics of the parental and chimeric viruses, a 50% egg infectious dose (EID_50_) assay was performed using 9-day-old SPF chicken embryos. Isolated viruses were inoculated into embryos at 100 EID_50_/0.1 ml. Six embryos were randomly selected at each time point including 24, 48, 72, 96, 120, and 144 h after inoculation. The allantoic fluid was collected and homogenized by pipetting, and EID_50_ was measured to determine the growth characteristics of viruses in chicken embryos.

To further check the stability of parental and chimeric viruses, viruses were successively passaged 20 times through embryonated eggs at 4-day intervals. The EID_50_ values of virus from the 1st, 5th, 10th, 15th, and 20th passage were then measured. Moreover, to determine if the P genes of these viruses harbored undesired mutations, total RNA was extracted from infected embryos and subjected to RT-PCR to amplify the region covering the P gene.

### 2.5. Pathogenicity Studies

The pathogenicity of recombinant chimeric viruses was determined by assessing the EID_50_, mean death time (MDT) in 9-day-old embryonated SPF chicken eggs and intracerebral pathogenicity index (ICPI) in 1-day-old SPF chickens as described in a previous report [23].

### 2.6. Thermostability Test

To determine the thermostability of the parent and recombinant strains, virus at 2 × 10^8^ EID_50_/0.1ml was incubated at 56°C for 60 min. One aliquot was taken every 10 min and stored at −20°C. The HA activity and EID_50_ were determined for all heat-treated viruses according to a previous report [17].

## 3. Results

### 3.1. Construction and Recovery of NDVs

To investigate the role of the P gene in NDV thermostability, we constructed chimeric cDNAs to exchange the P gene between the thermotolerant NDV4-C strain and the thermolabile LaSota strain. Sequence analysis of the chimeric cDNAs confirmed the successful exchange of the expected P gene with no undesired mutations.

The parental viruses rNDV4C and rLaSo and the chimeric viruses rNDV4CLaSoP and rLaSoNDV4CP were recovered and passaged according to previous reports [12, 14]. To confirm exchange of the P gene in these viruses, total RNA was extracted from infected chicken embryos and subjected to RT-PCR for amplification. Sequencing results confirmed successful exchange of the P gene.

### 3.2. Exchange of P Gene Has No Significant Effect on Growth Kinetics and Passage Stability of Recovery NDVs

**Figure 2:**
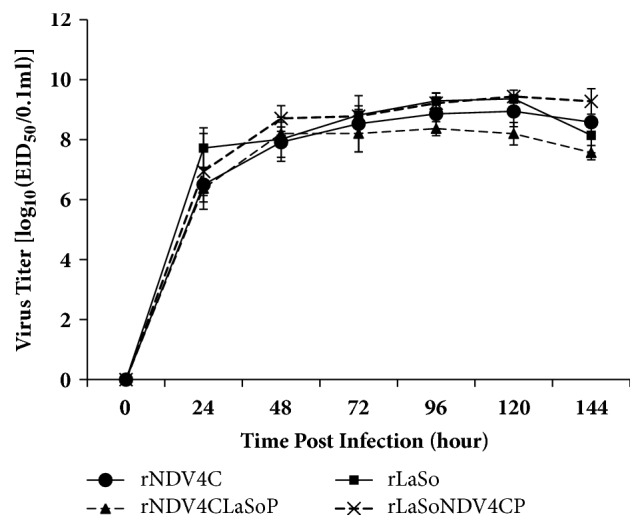
**Growth kinetics of chimeric and parental viruses in chicken embryos.** Recovery viruses were inoculated into the allantoic cavities of 9-day-old SPF chicken embryos at 100 EID_50_/0.1ml, and six chicken embryos were randomly selected at each time point including 24, 48, 72, 96, 120, and 144 h after inoculation. The allantoic fluid was collected and homogenized, and EID_50_ values were measured to determine the growth characteristics of viruses in chicken embryos.

To determine whether exchange of the P gene influences the growth kinetics of isolated NDVs, the EID_50_ values of chimeric viruses rNDV4CLaSoP and rLaSoNDV4CP harboring heterologous P genes were compared to those of the parental viruses rNDV4C and rLaSo. All viruses were inoculated into embryos to measure EID_50_. As shown in Figure 2, the titer of the parental rNDV4C virus increased until 96 h after inoculation and peaked at 120 h, after which it declined at 144 h. Similarly, the titer of the chimeric rNDV4CLaSoP virus harboring the P gene of the LaSota strain increased until 96 h; moreover the titer decreased at 120 and 144 h but was not significantly different compared to that of the parental virus. Meanwhile, in terms of growth characteristics, we found that the chimeric rLaSoNDV4CP virus harboring the P gene of NDV4-C strain, like the parental rLaSota virus, increased until 120 h and decreased at 144 h. The titer of rLaSoNDV4CP was slightly higher than that of the parental virus rLaSo and was approximately 0.6 log_10_ at 144 h (Figure 2).

**Figure 3:**
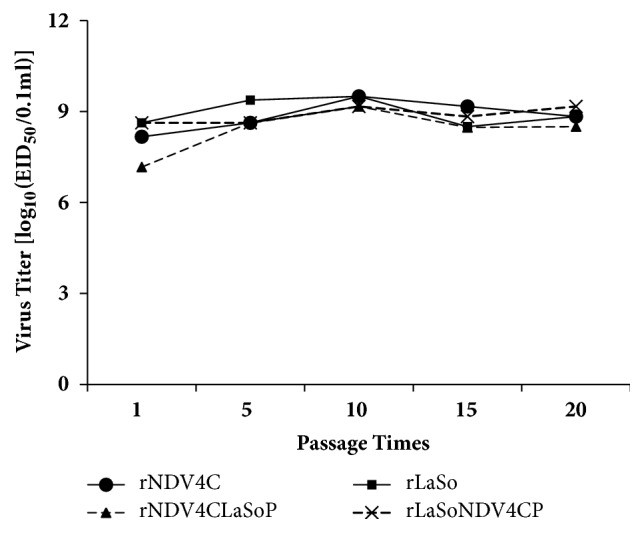
**Passage stability of recovery viruses. **All recovery viruses were continuously passaged using 9-day-old embryonated eggs, 20 times at 4-day intervals. The EID_50_ values were measured after the 1st, 5th, 10th, 15th, and 20th passage of the viruses.

To determine whether exchange of the P gene affects NDV passage stability, these viruses were continuously passaged 20 times through embryonated eggs. We found that the EID_50_ of viruses were stable at the 1st, 5th, 10th, 15th, and 20th passages (Figure 3). Moreover, the P gene of these viruses did not harbor any undesired mutations based on sequencing of the PCR products. Taken together, the growth kinetics and passage stability of the rNDV4CLaSoP and rLaSoNDV4CP chimeric viruses harboring the heterologous P gene are similar to those of the rNDV4C and rLaSo parental viruses, respectively, indicating that exchange of the P gene has no significant effect on NDV growth kinetics and passage stability.

### 3.3. Exchange of P Gene Does Not Alter the Virulence of Recovery NDVs

To investigate the effect of exchanging the P gene on virulence, the pathogenicity of rNDV4CLaSoP and rLaSoNDV4CP chimeric viruses harboring heterologous P genes was compared to that of rNDV4C and rLaSo parental viruses by performing MDT assays using 9-day-old chicken embryos and ICPI assays with 1-day-old chickens. Results showed that all chimeric viruses remained avirulent, similar to parental viruses, with MDTs >120 h and ICPI values of 0 (Table 1). Overall, all isolated viruses retained their lentogenic pathotype, implying that exchange of the P gene does not affect virulence.

### 3.4. P Is the Important Thermostable Determinant

**Figure 4:**
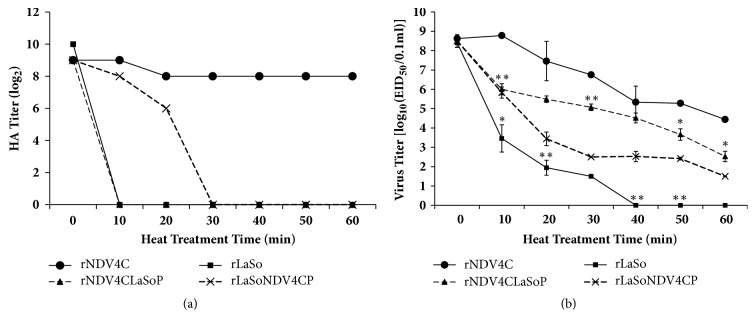
**Thermostability of parental viruses (rNDV4C and rLaSo) and chimeric viruses (rNDV4CLaSoP and rLaSoNDV4CP). **Heat-inactivation kinetics of HA activity (a) and virus titer (b) of the indicated recovery NDVs were determined at 56°C.* P* < 0.05 and* P* < 0.005 are presented as *∗* and *∗∗*, respectively.

To explore the effect of P on NDV thermostability, this gene was exchanged between the thermostable NDV4-C strain and the thermolabile LaSota strain. Heat resistance of the chimeric and parental viruses was evaluated by testing HA activity after high-temperature treatment. As shown in Figure 4(a), the HA activity of rNDV4C was maintained from 10 to 60 min, whereas the P gene of the thermolabile LaSota strain caused rNDV4CLaSoP to lose thermostability. Meanwhile, HA activity in the rLaSota strain suddenly declined to 0 after 10 min; however, the P gene of the thermostable NDV4-C strain prolonged the decline in HA activity in the rLaSoNDV4CP strain (Figure 4(a)).

Furthermore, heat resistance was evaluated by testing viral titer after high-temperature treatment. As shown in Figure 4(b), rNDV4C titer remained stable within 10 min, declined at a speed of 1 log_10_/10 min between 10 and 40 min, and decreased steadily from 40 and 60 min, obviously indicating wild-type thermostability. In contrast, the titer of rNDV4CLaSoP harboring the P gene of the thermolabile LaSota strain decreased strikingly by approximately 2.5 log_10_ within 10 min; moreover, the titer was 0.8–2.1 log_10_ less than that of the parental rNDV4C virus from 20 to 60 min. Hence, heterologous P gene expression reduced the thermostability of rNDV4CLaSoP. Moreover, we found that the titer of rLaSo declined sharply, by more than 5 log_10_ values in 10 min, and decreased approximately 1.5 log_10_ from 10 to 30 min and to 0 between 40 and 60 min. This indicates that this strain apparently retained the thermolabile characteristics of the wild-type strain. By comparison, the titer of rLaSoNDV4CP, harboring the P gene of thermostable NDV4-C, was 0.9–2.5 log_10_ values greater than that of the parental rLaSo virus from 10 to 30 min and maintained 1.5–2.4 log_10_ values from 40 to 60 min (Figure 4(b)). Therefore, heterologous P gene enhanced the thermostability of rLaSoNDV4CP. Taken together, we concluded that the P gene is the vital determinant of NDV thermostability.

## 4. Discussion

ND, caused by NDV, is highly infectious and pathogenic to many different types of poultry and results in serious economic losses to the poultry industry [2, 3]. Some thermostable NDV vaccines have been used because they are not dependent on cold chain for transport and storage. Nevertheless, the molecular mechanism of NDV thermostability is poorly understood. In the present study, we exchanged the P gene of the thermolabile NDV4-C strain with that of the thermolabile LaSota strain to explore the role of this gene in heat resistance. Our results demonstrate that P contributes to the thermostability of NDV.

P, F, HN, and L of NDV play roles in NDV virulence [13–16]. Wen et al. verified that HN, rather than F and L, is a critical determinant of NDV thermostability [17]. Meanwhile, Wen et al. also showed that the combination of NP, P, and M does not alter thermostability; however, the relationship between P alone and thermostability was not studied. It is possible that NP and M compromise the effect of P on this process, and thus, this relationship was not identified in their report.

A previous report indicated that the V protein (nonstructural proteins V and W, produced via mRNA editing of the P gene) of NDV is associated with viral pathogenesis [13]. In this study, we demonstrated that exchange of the P gene does not alter virulence, as both NDV4-C and LaSota strains are avirulent [12]. Interestingly, the titer of the chimeric rNDV4CLaSoP virus harboring the P gene of LaSota strain was approximately 0.8 log_10_ less than that of the parental rNDV4C virus at 120 and 144 h, and the titer of rLaSoNDV4CP was slightly higher than that of rLaSota and was approximately 0.6 log_10_ at 144 h (Figure 2). We hypothesize that because chimeric viruses were maintained at 37°C for a long time, P of the thermolabile LaSota strain caused the virulence of rNDV4CLaSoP to diminish, whereas P of the thermostable NDV4-C strain enhanced rLaSoNDV4CP virulence, which is consistent with our conclusion that P contributes to the thermostability of NDV.

The results of thermostability tests illustrate that, after heat-treatment, the kinetics of rNDV4CLaSoP HA activity were analogous to those rLaSo, whereas the HA activity of rLaSoNDV4CP was disparate from that of rNDV4C (Figure 4(a)). These data indicate that viruses containing homologous P genes do not exhibit the same thermostability. This is better reflected by the results of Figure 4(b), wherein rNDV4CLaSoP and rLaSo, or rLaSoNDV4CP and rNDV4C, harbor the same P gene, but the heat resistance was similar but not identical. We consider that other genes might also affect NDV thermostability, which is validated by the conclusions of the report by Wen et al. [17].

## 5. Conclusions

In summary, based on reverse genetic technology, we revealed that exchanging the P gene does not have a significant effect on growth kinetics, passage stability, or virulence of NDV. Moreover, P plays an important role in the thermostability of NDV. Our study contributes to the understanding of the mechanism underlying NDV thermostability and could form the basis for the use of the NDV4-C strain as a thermostable vaccine.

## Figures and Tables

**Table 1:** Biological characteristics of the recovered NDVs.

Virus	Pathogenicity		Titer in allantoic fluid	
	MDT	ICPI	log_10_(EID_50_/0.1ml)	HA titer (log_2_)
rNDV4C	>120 h	0.00	8.625	9
rLaSo	>120 h	0.00	9.375	10
rNDV4CLaSoP	>120 h	0.00	8.625	9
rLaSoNDV4CP	>120 h	0.00	8.625	9

## Data Availability

The data used to support the findings of this study are available from the corresponding author upon request.
